# The Radiology Virtual Reading Room: During and Beyond the COVID-19 Pandemic

**DOI:** 10.1007/s10278-021-00427-4

**Published:** 2021-02-23

**Authors:** Joseph H. Yacoub, Carl E. Swanson, Ann K. Jay, Cirrelda Cooper, James Spies, Pranay Krishnan

**Affiliations:** Medstar Georgetown University Hospital, 3800 Reservoir Rd NW Washington, 20007 Georgetown, DC USA

**Keywords:** COVID-19, Reading room, Virtual consults, Teleradiology, Imaging 3.0

## Abstract

The COVID-19 pandemic has disrupted the radiology reading room with a potentially lasting impact. This disruption could introduce the risk of obviating the need for the reading room, which would be detrimental to many of the roles of radiology that occur in and around the reading room. This disruption could also create the opportunity for accelerated evolution of the reading room to meet the strategic needs of radiology and health care through thoughtful re-design of the virtual reading room. In this article, we overview the impact of the COVID-19 pandemic on radiology in our institution and across the country, specifically on the dynamics of the radiology reading room. We introduce the concept of the virtual reading room, which is a redesigned alternative to the physical reading room that can serve the diverse needs of radiology and healthcare during and beyond the pandemic.

## Introduction

The COVID-19 pandemic has been a major and rapid disruptor to life and work around the world. The concept of social distancing, as a way of containing the spread of the disease, has altered many aspects of life, and particularly in healthcare. In radiology, the unexpected sudden need for social distancing has disrupted the workflow of the traditional reading room. The duration of the interruption to the routine workflow in radiology reading rooms remains uncertain, but it is unlikely that after a prolonged disruption, it will ever return to its previous state. The radiology field could be standing at a bifurcation in the road, choosing between abandoning the reading room or evolving it. Through the thoughtful application of informatics, the concept of the radiology reading room can evolve to meet the future needs of radiology and the healthcare system during and beyond the pandemic. In this article, we will discuss how social distancing can force an accelerated evolution of the concept of the reading room, into a new concept of the virtual reading room.

### The Traditional Reading Room

The radiology reading room is a central concept in radiology that has evolved with the evolution of the field. It can be loosely defined as the physical space in which radiologists perform their duties, typically providing coverage as a group, and where they are accessible to other healthcare providers in-person and via phone. The design and layout of the reading room evolved in the film-based era and persisted with limited modification in the PACS era in many institutions [1]. The transition to PACS around the turn of the century was a major disruptor to the dynamics of the reading room [2]. With radiology images being widely accessible from outside of the radiology department, there was less need for referring physicians to visit the reading room [2]. Despite the disruption, the radiology reading room continues to get frequent phone calls and visits from various health care providers in many institutions, with phone calls as frequently as every 4 min in one study [3]. In the modern PACS era of radiology, many hospital-based radiology departments, particularly academic departments, continue to emphasize the accessibility of the reading room [4].

The introduction of PACS has also enabled teleradiology, which has since become widely used and has been shown to be effective [5]. A survey in 2007 estimated that 40% of radiology practices have performed outside reading which accounted for 11% of their workload and about 4% of the workload of all radiologists [6]. After reviewing the literature in the decade from 2005 to 2015, Bashshur et al. concluded that the practice of teleradiology is well established, widely accepted and effective [5]. While the benefits of teleradiology are widely acknowledged, the on-site reading room remains the epicenter of radiology activity in most academic institutions and many community institutions. The ACR task force on teleradiology is clear that “on-site coverage is preferred,” with teleradiology service ideally used as supplemental to a comprehensive on-site radiology practice [7].

The importance of the reading room and its survival despite the alternative of teleradiology stems from the fact that the reading room hosts several activities beyond the interpretation of examinations. A thoughtful redesign of the reading room needs to consider all these activities, namely:

• Interpretation of examinations
• Collaboration among radiologists (e.g., second opinion, interesting case sharing, feedback and follow up)
• Communication with radiology team: radiology technologist and sonographers (e.g., protocoling and checking studies)
• Resident education (e.g., resident readouts, interesting cases)
• Multidisciplinary conferences (while they do not typically happen in the reading room, they are a natural extension of the reading room and therefore discussed in this article)
• Consultation for referring physicians
• Patient consultation (currently limited to breast imaging and interventional radiology, but with an increasing interest in the broader radiology community)

In the PACS era of radiology, the phone has become a natural extension of the radiology reading room, whereby many of the above activities happen through the radiology reading room phone.

### Radiology Value Propositions

The design and function of the radiology reading room is not merely a question of technology and technical feasibility, but it should be primarily about serving the strategic goals of radiology. While currently, volume and metrics based on Relative Value Unit (RVU) remain the primary measures of productivity, there is an expected shift towards value-based metrics. Reforms in physician reimbursement and newer models such as accountable care organizations and bundled payments might further drive the shift towards measuring value and shifting away from a focus on the volume of studies performed [8, 9]. Radiology departments will have to give more consideration to their value propositions, where “value” would include things such as quality, service, resource management, and professional development [10] (Table 1). Many value-added activities performed by radiologists in and around the radiology reading room are investments that do not directly translate into billing revenue, at least not at the current time; however, they are critical for the survival and progress of the field. To disregard such value-added activities would turn the radiologist’s work into a commodity, differentiated only by price. Furthermore, over the past decade, radiology organizations and societies have emphasized the need for increasing the visibility and accessibility of radiology to combat the commoditization of radiology. With competing pressures for productivity and value, it will be increasingly important to not only encourage value-added activities but to also measure, track, and promote them. This increasing focus on value is at the core of the ACR Imaging 3.0™ initiative, which includes a broad set of initiatives addressing the visibility of radiologists and emphasizing the role of radiologists in managing all aspects of imaging care to improve patient safety and outcomes and to deliver high-value care [11–13]. From another perspective, radiology has long led the rest of medicine in the digital age, and the field must continue to lead in applying ongoing innovation in information technology and communication systems in supporting new approaches to knowledge management and communication [14]. At the heart of the Imaging 3.0 initiative is empowering radiologists to leverage technology to deliver value [11–13].

**Table 1:** Examples of value-added activities occurring in and around the reading room categorized based on value-added matrix developed and described by Patel S[10]

Value-added category	Definition	Examples of value-added activities in the reading room
Quality	Activities related to quality assurance, quality control (QC), and patient safety	Reporting quality, peer review/peer learning, second opinions, multidisciplinary conferences, protocol management, technologist staff feedback, QC of imaging studies
Service	Activities performed to satisfy a need or fulfill a demand often in the context of interaction with patients or referring physicians	Critical results management, referring provider communication/consultations, improving turnaround time, subspecialty accessibility, patient supervision, patient consultations and overall contributing to positive patient experience
Resource management	Efficient utilization of resources including equipment, supplies and personnel	Evaluation of appropriateness and medical need for studies, efficient utilization of radiologist schedules
Professional development	Activities relating to acquiring skills and knowledge and career advancement	Teaching

## The Virtual Reading Room During and Beyond COVID-19

Social distancing during the COVID-19 pandemic had an immediate impact on the radiology reading room. This was accompanied by a rapid decline in volume of studies that occurred in the initial few months of the pandemic and normalized slowly overtime. At our institution and many around the country, alternatives were rapidly devised to avoid having radiologists crowded in a limited space. In this section, we describe the alternatives that were developed in response to social distancing. Our practice represents the academic division of a larger radiology group serving one of the largest healthcare providers in the region. Our system includes one academic hospital, nine community hospitals, and many outpatient facilities including multiple imaging centers. The entire radiology practice is supported by a unified PACS network across all hospital and outpatient facilities. Our discussion will be more focused on the academic practice of the enterprise which is composed of 43 radiologists, 24 residents, and 6 fellows who, before COVID, provided onsite coverage to the academic hospital, an affiliated community hospital, and two imaging centers. We will refer to the broader radiology practice where applicable.

### Interpretation of Examinations

#### During the Pandemic—Interpretation of Examinations

Interpretation of examinations and rendering reports is the quintessential activity occurring in the reading room. It is also the activity that has been proven to be done effectively remotely [5]. Decades of success with teleradiology made the rapid transition to remote interpretation feasible, though not without challenges. Our larger radiology group had long supported home and cross-site reads given the wide geographic spread of our enterprise and the use of remote radiology for overnight reads in the non-academic practice of our enterprise. Nearly all radiologists in the non-academic practice were already equipped with home workstations. In our academic practice, however, very few radiologists had home workstations. This difference between the academic and non-academic practice was due to the difference in the culture of the practices and the decisions of the radiology group leadership and not due to technical factors since the informatics infrastructure was unified. Before the report of the first COVID-19 case in our area, the leadership of our academic radiology practice recognized the pending need for equipping the academic radiologists with home workstations. Communication with other institutions around the country was critical at this phase due to the novelty of the situation in North America. Limited knowledge of the experience of some Asian countries with the severe acute respiratory syndrome (SARS) outbreak in 2003 was all the experience available in responding to pandemics of such magnitude [15]. However, despite the early recognition of the potential needs of the academic practice, it took several weeks to complete the deployment of additional home workstations. During this gap, we had to resort to several temporizing strategies to mitigate the risks which are described briefly here. One strategy was team segregation in which radiologists from one subspecialty were divided among separate physical reading rooms so that if an exposure happens in one of the physical reading room requiring quarantine, the impact would not all fall on a single subspecialty that would stall the clinical operations. Another strategy was reducing the on-site staffing of attending and resident radiologists to the minimum required. The rapid decline in the volume of imaging studies allowed for continued operation with reduced staff. Approximately 2 weeks after WHO’s characterization of COVID-19 as a pandemic, workstations were issued for all the radiologists in the academic practice except for breast imagers and interventional radiologists. Reducing the staff and enabling remote reads allowed us to decompress the reading rooms, thereby reducing the occupancy to less than half pre-pandemic capacity. In general, a team of one attending radiologist and one resident with or without one fellow provided on-site coverage for each of our 3 primary reading rooms and one attending provided onsite coverage for our community hospital. This translated into each radiologist providing on-site coverage on average about once a week, with some subspecialty sections preferring to alternate coverage daily and other alternating coverage weekly. After the peak of COVID-19 cases had been passed and with the gradual return of imaging volumes closer to pre-pandemic volumes, we increased the onsite coverage by adding one more attending and one resident for busier reading rooms. By that time, more rooms and offices had been repurposed as reading rooms further decompressing the reading room and providing ample social distancing between the onsite staff.

We faced two main supply chain issues in acquiring additional workstations and monitors: unplanned/unbudgeted expense at a time where declining volume called for vigilant fiscal responsibility, in addition to massive shipment delays. The diagnostic quality monitors our institution generally sources are manufactured and shipped from northern Italy, which was one of the initial hotspots of the COVID-19 pandemic. To respond to these supply chain challenges, we partnered with our enterprise IT colleagues to create a new home workstation build that balanced a standardized hardware deployment with hardware available from a supply chain perspective. The workstations we deployed included medical grade diagnostic monitors that conformed to the ACR standards in resolution, luminance, and calibration. An alternative to medical grade monitors is commercially available, non-medical grade monitors, with at least 4 megapixel resolution (pixel pitch ≤ 0.2 mm) and luminance of at least 350 cd/m [2] that meets the resolution and luminance requirement ACR technical standards [16, 17]. However, these do not conform to the grayscale calibration requirements of the ACR and additional steps of generating and installing DICOM grayscale look up tables as well quality control check of the calibrated monitors would have to be performed. Open source software packages are available to perform these steps [18].

Recognizing that the response to COVID-19 caused additional clinical and administrative roles beyond radiology to move outside of the hospital and the internal network, we again partnered with our IT colleagues to analyze the readiness of the network and PACS system. They determined that the VPN (Virtual Private Network) would become quickly saturated as local workflows were transitioned remotely and took two actions: increased the available bandwidth of the existing VPN by a factor of 5 and created a dedicated Imaging VPN. We analyzed the compression strategy on the PACS and determined that only lossless compression was utilized and ensured this was enabled for all remote interpretation.

Similar efforts of remote reading and decompressing the reading room have been reported across the country [19, 20]. Quraishi et al. surveyed 72 academic practices and 102 community practices across the country that were affiliated with a radiology residency program. They reported that nearly three-quarters of practices switched normal daytime shifts to internal teleradiology, with a similar percentage for academic and community hospitals. About a third of the surveyed institutions had to increase the provision of home workstations to support this shift in practice, with more of the academic practices having to do that [21]. In this survey, 45% of the community practices and 25% of the academic practices, left their reading rooms unstaffed [21]. This shift to remote reading has been reported and encouraged by multiple reports during the pandemic [4, 19, 20, 22]. As for on-site coverage, models of decompressing the reading room and creating single-radiologist and single-workstation reading rooms have been reported and encouraged [19, 22].

#### Beyond the Pandemic—Interpretation of Examinations

The implication of this widespread and rapid shift to home interpretation at our institution and across the country is worth careful examination. Continuation of the policies and strategies of social distancing that evolved during the pandemic is likely to continue for at least the medium term, including decompressed reading rooms or if possible single workstation reading rooms and home interpretation [23]. Furthermore, over half of the respondents of the above-mentioned survey reported that they perceived enough benefit from their experience with “internal teleradiology” that they plan to continue a similar workflow after the pandemic subsides (64% of community radiologists and 46% of academic radiologist) [21]. These reports coupled with the prolonged duration for the social distancing, strongly indicate that the disruption to the traditional reading room with remote reading and fragmented reading rooms will persist for a long while and will outlive the pandemic. Some have suggested that remote reading may also allow practices to expand radiologist hours providing increased flexibility in scheduling [24]. In summary, providing remote interpretation is perhaps the one function of the reading room that most radiologists would agree can be performed equally well on-site or off-site when considered in isolation. It is the other functions of the reading room that need more careful consideration.

### Collaboration Among Radiologists

#### During the Pandemic—Collaboration Among Radiologists

**Fig. 1:**
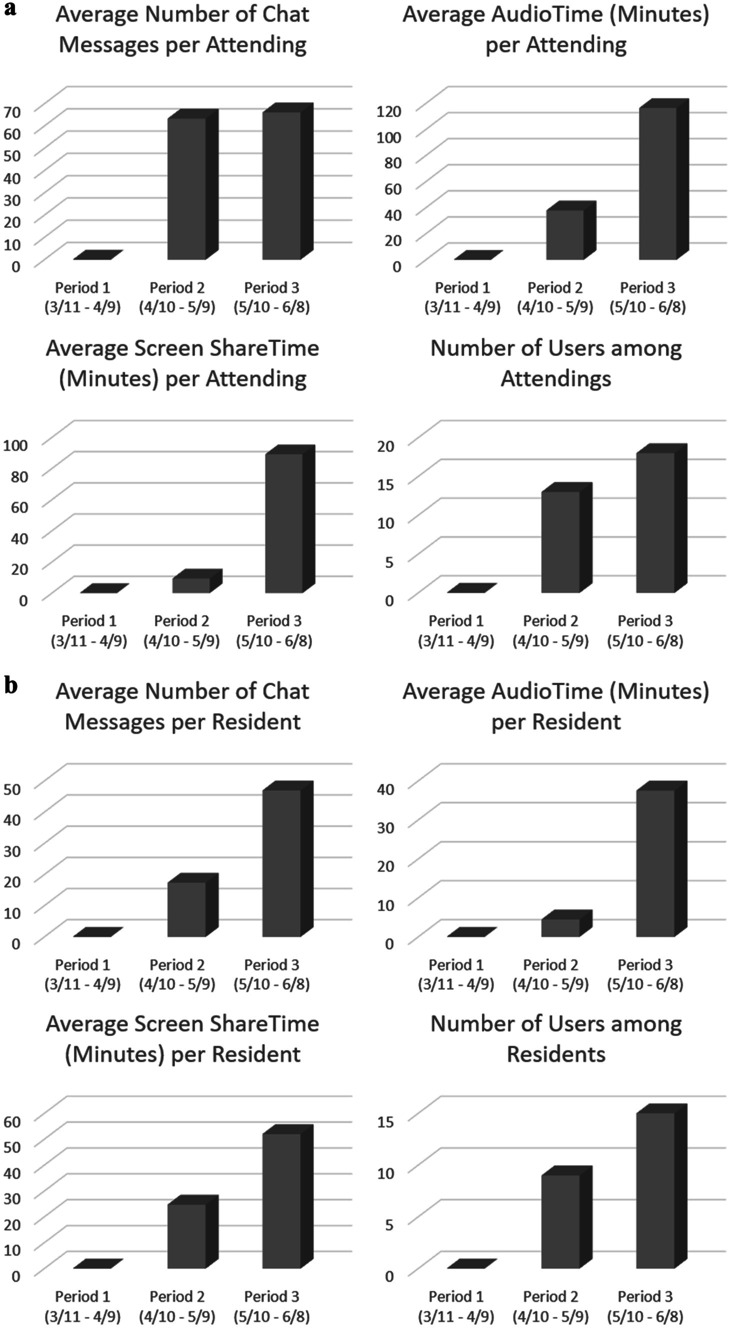
Usage data among the radiology attendings (**a**) and radiology residents (**b**). Period 1 is the period from the characterization of COVID-19 as a pandemic until the start of the use of MS Teams in the department (March 11 through April 9). MS Teams was available but was not in use during this period. Period 2 is the following 4 weeks representing the early adoption of MS Teams (April 10 through May 9). Period 3 is the subsequent 4 weeks representing increasing adoption by attendings are residents (May 10 through June 8)

The radiology reading room has traditionally provided a natural environment for collaboration among radiologists. At our institution, the practice of seeking second opinions or sharing interesting and instructive cases is an integral part of our group dynamics. Continuing communication among the radiologist is critical to preserve, particularly given the novelty of the situation that required frequent communication. The natural alternatives to in-person communication were mobile phone messaging (SMS) and more reliance on email communications; however, SMS was not designed for professional team communication and was not suitable for discussion of cases that include protected health information (PHI). At about 5 weeks into the pandemic, we transitioned to an online collaboration software called Microsoft Teams (MS Teams®, Microsoft, Redmond, Washington, USA), which was fortunately already available across our enterprise and was preinstalled and configured on all new home workstations and several of our existing on-site workstations. MS Teams was configured in our enterprise to be HIPAA compliant which allowed for discussions that include PHI [25]. While it represented a shift in the method of team communications that needed some adjusting and education, within a few weeks it gained acceptance in the body imaging section that piloted its use. The Teams feature of the software allowed us to recreate our morning huddle virtually and provided a venue to share cases and carry on discussions. Preliminary data of our initial pilot in the abdominal imaging section, which is composed of 10 radiologists, demonstrated that there were 44 posts, 78 replies, and 58 mentions in the last 30 days (including weekends) to our “morning huddle” channel. The chat feature allowed for one-on-one communication such as seeking second opinions and giving feedback. The usage date for the chat feature as well as voice communication and screen sharing are depicted in Fig. 1 and will be discussed further as we describe its use in resident education. Meanwhile, our bi-weekly section meeting continued via Webex® (Cisco, San Jose, California, USA).

Similar online collaboration tools for team communication have been described, with multiple modern PACS systems providing built-in messaging tools, which provide similar chat functions. These features are popular among radiologists. Another alternative to MS Teams that has widespread use in the information technology world is Slack® (Slack Technologies, San Francisco, CA, USA) which can also be configured to be HIPAA compliant [26]. There is no published literature on the use of MS Teams or Slack in radiology to our knowledge; however, through informal communication, we are aware of other radiology societies or departments that started the use of these tools, which may represent the early stages of adopting group chat tools among radiologists. Several additional group chat solutions exist some of which offer HIPPA compliance but there are no reports of their usage in radiology to our knowledge. The use of these electronic communications tools is generally encouraged during the epidemic and in the absence of these tools, direct phone communication is certainly preferred over in-person communication [19].

#### Beyond the Pandemic—Collaboration Among Radiologists

Before the pandemic, many collaborative efforts in the reading room occurred informally and spontaneously, while some were somewhat more structured in the form of “the case of the day” or “a morning huddle.” With home interpretation predominating during the epidemic and likely persisting for a long time, recreating the team dynamic via communication technology should be a priority. Losing this team dynamic may have a long-term impact on professional satisfaction and development that will be hard to measure and quantify. Empirical studies on this topic are limited in the radiology literature. Perhaps we can illustrate by extrapolating from one well-studied aspect of radiology collaboration, namely peer learning. The most common form of peer review is the RADPEER system developed by the ACR in 2002 which reflects a traditional quality assurance approach, derived from manufacturing in the mid-1900s [27, 28]. Despite its wide adoption, multiple recent articles have highlighted the limited-value of the system [27–30] with recent calls for a different approach, namely peer learning [27, 28]. Peer learning emphasizes collaborative learning environments and interpersonal professional relationships [28, 31, 32]. This transition from peer review that focused on impersonal quality assurance to peer learning, which is deeply collaborative, stresses the need for maintaining collaboration in the reading room. Modern technology companies have taken the concept of collaboration to new levels, by designing physical office spaces and collaboration software solutions, that promote collaboration and open communication [33, 34]. This collaboration is inherent in the radiology reading room.

The advantages of collaboration tools like MS Teams and Slack are that, in addition to their direct messaging feature, they recreate a team dynamic. Our experience during the pandemic has been promising in this regard. Our pilot of using MS Teams to maintain a virtual team dynamic among radiologists has been successful and we are now in the process of expanding it by including residents and expanding to other sections. This practice of virtual collaboration using electronic media is expected to continue to thrive post the pandemic even with increasing on-site coverage. The fragmentation of the physical reading room that happened during the epidemic will likely enshrine the reliance on electronic communication. One benefit that these tools have over face to face or phone communication is that they enable asynchronous communication with the ability to escalate to synchronous communication when needed. Using it in such a way can help reduce interruption to concentration. It also gives the radiologist more control over availability to respond to requests when engaged in attention intensive tasks. The ease of use of these asynchronous modes of communication may encourage increased collaboration. These tools would eliminate barriers between subspecialty reading rooms, possibly encouraging more collaboration across subspecialties of radiology.

### Communication with the Radiology Team: Radiology Technologists and Sonographers

#### During the Pandemic—Communication with the Radiology Team

Radiology technologists and sonographers (collectively referred to as RTs in the rest of the article) are an integral part of the radiology team. According to the ACR task force on teleradiology, RTs must function under the supervision of a qualified licensed physician [7]. Therefore, maintaining communication between radiologists and RTs is critical. This communication pertains to protocoling of examinations, reviewing studies, and handling patients’ inquiries and medical needs. In our institution, an electronic protocoling tool was already in use for several years which streamlined a significant portion of communication with technologists and which allowed for a smooth transition when we implemented social distancing. Most of the remaining communication needs and support were provided by at least one on-site radiologist for each subspecialty. On-site trainees also helped to provide RT support under the guidance from the on-site radiologist. Even before the pandemic, the large size of the hospital meant that the phone served as an extension of the reading room with many RTs communicating with radiologists via phone. The exception to that was sonographers who reviewed their cases in person. During the pandemic, we shifted most of these communications to the phone. We tried to decrease the demand on the limited on-site staff by directing some of these calls to home readers, but for all practical purposes, the on-site staff fronted most of the calls. A similar approach predominates in many departments across the country [19]. It is important to note the absence of radiologists who were working remotely at a time marked by increasing stress levels among healthcare workers, created an unanticipated tension between the RTs and the radiologists.

#### Beyond the Pandemic—Communication with the Radiology Team

Beyond the epidemic, we anticipate a role for asynchronous tools for communication between RTs and radiologists, which could be more efficient and less disruptive to the workflow of both groups. These tools will be beneficial irrespective of the physical location of the radiologists. As we discuss in the section on consultations below, replacing the radiology reading room phone with an asynchronous tool that allows radiologists to function as a team despite their geographic dispersion will be at the heart of recreating a virtual version of the radiology reading room. The on-site presence will still be important to provide support to the RTs and handle occasional patient safety considerations (e.g. contrast complications); however, this on-site presence can be handled by a smaller staff, once the necessary supportive culture has been built and communicated.

### Resident Education

#### During the Pandemic—Resident Education

The radiology reading room is where resident education primarily happens. One of the biggest concerns and challenges of social distancing during the pandemic is resident education, particularly in the reading room. The requirements for social distancing and government-issued sheltering-in place mandates caused significant disruptions to the education of our residents. Eighty percent of our residents were required to shelter at home with instructions to immerse themselves in “independent learning.” While independent learning was more feasible for the more senior residents, this was much more difficult for the junior residents. The first-year residents were the most vulnerable group affected by the pandemic. The second half of the academic year is when they start their second rotations in the different subspecialties, with the goal of honing their skills to prepare for taking independent call starting July 1. Furthermore, with the majority of attendings reading from home on any given day, even the on-site residents were at a disadvantage. To alleviate the latter problem, we initiated the use of MS Teams to do virtual readouts. The chat feature allowed for rapid communication and feedback and the screen-share function proved to be valuable in creating a virtual readout experience comparable to the side-to-side readout. The integration of the software in the workstation reduced the overhead of adoption. Our usage data of MS Teams for radiology attending and radiology residents are shown in Fig. 1.

Similar efforts in scheduling resident and remote readouts were reported around the country. Some programs scheduled residents on a 1-week-on, 1-week-off cycle [35]. Some described the use of PACS integrated messaging tools to communicate with residents [4]. Multiple reports described the use of teleconferencing to perform remote readout with screen sharing [4, 22, 35, 36].

The other aspect of resident education was didactic education that typically occurred outside of the reading room. This was particularly important as a large percentage of residents were sheltering at home and as the volume of cases plummeted. We, therefore, increased the amount of didactic education and case conferences. All resident conferences were virtual via Webex, which proved to be effective, with excellent resident participation. Attendings reading from home were to continue and intensify their didactic education efforts during the pandemic. Additionally, there was a tremendous national and international effort to quickly create and offer free educational content for all residents sheltering at home. World-class educators offered free lectures and multiple radiology societies, such as the Association of Program Directors in Radiology (APDR), created curricula on a scheduled basis. Given that all lectures were virtual, there were numerous cross-institutional collaborations to provide additional educational content that was provided to residents in addition to our usual lecture curriculum provided by our faculty. To address the specific issue of the first-year residents, many faculty held additional case conferences for this class alone, to target must-know call cases. While the response of the academic radiology community was remarkable, the residents and faculty alike came to understand the meaning of “Zoom burnout.” Despite being a specialty that sits in front of a computer all day, participating in hours of lectures online proved to be draining. Similar effects were reported across the country [4, 36].

#### Beyond the Pandemic—Resident Education

No studies have yet evaluated the relative educational value of live virtual readout with screen sharing to the traditional side-by-side readout; however, our early experience suggests that it is well received by residents and attendings. Beyond the pandemic, virtual readouts are likely to continue. With many academic institutions having a wider geographic spread with multiple hospitals and imaging centers, virtual readout may serve an increasing role and provide more scheduling flexibility. This may also be the case as physical reading rooms become more fragmented to allow for continued social distancing. However, department collegiality and opportunities for mentorship will have to be prioritized if remote readout continues beyond the pandemic [24]. As previously mentioned, we are in the process of including residents and fellows in the teams we created in MS Teams to recreate some of the dynamics of the reading room that residents traditionally participated in such as huddles as well as planned and spontaneous sharing of cases. Studies evaluating the relative educational value of virtual readout and the perceptions of attendings and residents are encouraged.

### Multidisciplinary Conference

#### During the Pandemic—Multidisciplinary Conference

Multidisciplinary conferences are a formalized form of radiology consultation and are a requirement for cancer centers and a valuable tool for providing the highest quality of care. Enabling radiologists’ participation in multidisciplinary conferences is critical. Before the pandemic, many conferences were already providing an option for teleconferencing for remote participants from satellite clinics, but, our radiologists always presented in person. During the pandemic, multidisciplinary conferences have transitioned very smoothly to almost exclusively virtual forms. The new virtual format has underscored the central role of the radiologist in the multidisciplinary management patients. This virtual format also enabled trainees to attend, which, is strongly encouraged particularly during the period of low imaging volumes.

#### Beyond the Pandemic—Multidisciplinary Conference

Multidisciplinary conferences have major implications on patient management [37], and specifically with refining the imaging findings and recommendations beyond the initial reports [38]. Fortunately, virtual multidisciplinary conferences have worked very effectively during the pandemic and could continue at least in part virtually. A common challenge when in-person multidisciplinary conferences were held outside of the radiology department was that the computer systems used were not optimized for PACS viewers, which often created an uncomfortable working environment for the presenting radiologist. With teleconferencing, the radiologist may have more control over the IT environment and the tools used. Advanced radiology software tools can easily be incorporated in the conference, such as 3D visualization tools, etc.… Teleconferencing also allows other radiologists who are not presenting to attend these conferences with more ease and can certainly give trainees more opportunities for attendance. On the other hand, the consequences of the loss of face-to-face interaction will be hard to measure and quantify. Radiology may have to learn from other industries that have already gone down this route.

### Radiology Consultation

#### During the Pandemic—Radiology Consults

The radiology reading room is the access point to radiology expertise, and it is this kind of access to expertise that is critical to preserve. In our institution, during COVID-19, in-person radiology consults have been replaced by phone consultation. Signs were placed on the reading room with phone numbers to call to access the radiologist. The on-site radiologist and trainee continue to field all calls to the reading room. Remote radiologists are available through their personal phones. A tool integrated into our worklist application provided a list of all actively reading radiologists and their phone numbers thereby making the redirection of incoming calls simpler. The list is only viewable to radiology users of the worklist application and is inaccessible to ordering physicians, thus maintaining the role of the reading room as a router for all incoming calls. Despite the signage, in-person consultation continued in the reading room. We have also attempted to provide virtual consults to ordering physicians via MS Teams with screen sharing. However, these efforts are still in their infancy and have not been systematically piloted yet. On the other hand, in the non-academic division of our practice, a radiology operator service has been in place for several years, termed Radiology Operation Center (ROC). During the pandemic, the ROC continued the routine role of fielding calls for radiologists across a wide geographic spread as well as aiding the radiologists in reaching the referring providers with critical results. Access to the ROC is integrated into our worklist application.

Around the country phone and telecommunication have been encouraged for virtual consults [19]. Academic departments that have encouraged in-person consultation had to give careful thought to consultation with respect to the pandemic [4]. Some have placed signage on reading rooms and information for the providers about means to access radiology via phone or messaging platforms [4, 39]. One IR division had an e-consultations (asynchronous provider-to-provider communications) tool in place, which was being considered for increased utilization [4, 9]. Another institution started hosting radiology virtual rounds with clinicians on the floor including radiology residents [36]. However, policies and procedures using the technology for greater clinician-clinician interaction over remote sessions are lacking in most institutions.

#### Beyond the Pandemic—Radiology Consults

The transition to PACS around the turn of the century has shifted the communication with referrers to electronic forms of communication [2]. Image accessibility outside of the radiology department resulted in many consults coming in the form of phone calls. Rapid turnaround times of reports also mean that an increasing portion of consults to radiology are seeking the radiologist expertise and insight as opposed to simply asking for preliminary interpretations, though the later practice still prevails in the emergency departments [40]. The radiology societies are emphasizing the need for radiologists to maintain meaningful relationships with ordering physicians. These relationships not only protect radiologists from being replaceable [8, 41] but, the absence of these relationships may undermine the radiologist's sense of professional dedication and fulfillment [8]. Referring physicians highly value these relationships and consider them important in developing interprofessional trust [42]. Furthermore, radiology consultations have management implications. One study found that 33.9% of consultations resulted in a new finding, a change in the severity of a previously detected finding, or a change in management recommendation [43]. It is therefore a matter of placing patient care first and has been strongly stressed by the ACR task force on teleradiology [7]. While one survey reported that only a minority of radiologists perceived less rapport with other physicians during the pandemic [21], radiology consultation is likely to be the area that suffers the most from remote reading if no solution is developed to make radiology more accessible. This should be at the heart of the re-design of the virtual radiology reading room.

Early models of systems and procedures to allow physician-to-physician subspecialty consultation in other branches of medicine have shown to be effective in providing care with high provider satisfaction [9]. In one instance e-consults were introduced in the context of accountable care organizations with the physician-to-physician e-consults replacing some of the direct patient subspeciality patients consults at a reduced fee [9]. In diagnostic radiology, Rosenkrantz et al. have described a system to allow referring physicians and radiologists to efficiently conduct instant-messaging based virtual consults that allowed for screen sharing [40]. In their study, referring physicians had a highly favorable response to the virtual consult system, indicating that it “tended to improve their understanding of the radiology report and to affect patient management, being particularly valuable in situations in which traditional consultation was difficult because of time or location restraints” [40]. On the other hand, while radiologists recognized the perceived value of the system for the referring physician, they found it disruptive to their workflow [40].

Unlike other subspecialties, diagnostic radiology imaging consultations are not billable events, yet they contribute value to patient care which is important to capture and measure. Creating a radiology virtual consult solution could make radiology even more easily accessible for referring physicians particular given the increasing sizes and geographic spread of many institutions. A radiology virtual consult system would also allow tracking and quantifying volume of consults provided by radiologists which would help estimate the value provided by radiology beyond the volume-based metrics of RVU and report turnaround time. This would align with the new era of radiology value propositions that emphasizes patient care and quality over volume.

For a radiology consult solution to be effective it needs to address the following 8 considerations:

1. It needs to be easily accessible to the referring providers

The ACR task force on teleradiology emphasizes that the methods of communication should be the choice of the referring provider [7]. If the system is not easily accessible and user friendly, it will simply not be used. Various access points to the system must be designed including apps for handheld devices and links from the hospital portals and the medical record system. The interface must be intuitive and easy to use. Consideration has to been given to the accessibility of this system by referrers within and outside the enterprise. The latter may introduce more challenges to the design of the system.

2- It needs to be well integrated into the radiologist workflow

The system needs to be built into the radiology workstation. Ideally, one interface would meet all the radiologist communication needs, whether with radiology colleagues, trainees, RTs, and referring providers. While, this may be difficult to achieve, at a minimum one interface should handle all alerts for incoming communications, even if the radiologist had to resort to other systems to respond to the communication (e.g. call back via phone).

3- It needs to allow the radiologist to function as a team

Inherent to the design of the virtual reading room is that radiologists can continue to function as a team despite of their physical separation. This provides continuity of care and accessibility around the clock. The ACR task force on teleradiology emphasizes that the radiologist should always be available for consultation with referring physicians [7]. This could only be achieved if the radiologists work as team, providing coverage for each other. The burden of tracking a specific radiologist who may or may not be on duty should not be laid on the referring physician. The physical reading room provided such continuous access to the radiologist by acting as a fixed access point to the radiology team. The virtual reading room should likewise allow referrers to direct their inquiries to a team of radiologists that is accessible around the clock.

4- It needs to be asynchronous but timely

Despite the importance of radiology consults, they can be a source of recurrent interruption to the radiologist’s workflow [44]. As previously mentioned, phone interruptions occur as frequently as every 4 min in one study of on-call radiologists. The on-call radiologist can expect to be interrupted 2 to 3 times during the interpretation of a routine CT abdomen and pelvis [3]. Another study found a correlation between phone interruption and error rates for on-call residents' preliminary reports [45]. Another study looking at the interruption in knowledge-intensive environments mitigated the effect of interruption by an operational policy of “sequestering,” where some service resources are protected from interruption [46]. A system that allows for asynchronous, yet timely communication, reduces the negative effect of constant interruptions. Consultation requests can be handled between cases as opposed to in the middle of cases, particularly when approached as a team, which will likely improve efficiency and reduce error. The system needs to be timely to maintain the trust of the ordering physicians. A turnaround of time 10 min or so for routine requests and shorter turnaround time for emergent inquiries can serve both purposes of timely responses and reduced interruption during examinations. These target turnaround times can be set based on surveys of referrers' expectations and needs.

5- It needs to allow the radiologist to respond to the consults effectively and efficiently

The ability to address simple requests via messaging integrated into the workstation can enhance the efficiency of the radiologist. More involved consults requiring image review can benefit from advanced features such as screen-sharing which can enhance the effectiveness of the consults. These advanced consult features could enhance the role and perception of radiology in regards to patient care. In the study by Rosenkrantz et al. screen sharing was used in 15% of virtual consults and was found helpful by 70% of referring physicians [40].

6- It needs to track the consults

Virtual consults can enable quantification of the noninterpretative work performed by radiologists. While consults are not RVU generating, they are critical in many respects and therefore need to be at least quantified and tracked. The ability to track the volume and nature of radiology consults will allow for quantification of the value-added by radiology beyond volume metrics.

7- It needs to provide the ability for limited rapid documentation

Unlike other subspecialty consults, radiology consults are often informal with only a minority of them being documented by the radiologist. A larger portion of these informal consults is documented by the consulting provider without the knowledge of the radiologist with room for misrepresentation and communication errors [43]. Authors of one study recommended that “Radiology practices should consider developing policies requiring radiologists to document informal consultations potentially affecting patient management, while developing solutions to facilitate such documentation when it is not readily achieved through report addenda (e.g., through direct documentation by the radiologist in the EMR)” [43]. A radiology consultation system could provide limited documentation capabilities for most routine simple consults. Consults that may have implications on patient management would still have to be documented in the radiology report or the electronic medical record. Won and Rosenkrantz state that “the importance of such documentation is highlighted by the observation that physician-to-physician communication errors are among the top reasons for medical malpractice claims in radiology” [43].

8- It needs to allow for intelligent automation

In the era of big data, natural language processing, and machine learning, digitizing consultation requests can allow for more intelligent routing of the requests and perhaps automation of some routine requests that do not need the radiologist expertise. A large number of phone calls to radiology do not require radiology expertise [44] and can be handled either by intelligent automation, other staff, or notification to the radiologist that can be rapidly acknowledged for the satisfaction of both requester and the radiologist.

### Patient-Centered Radiology

#### During the Pandemic—Patient-Centered Radiology

At our institution patient consultations are limited to breast imaging and IR. For the rest of diagnostic radiology, the question of direct patient consultation has not been an issue either before or during the pandemic. Around the country, efforts of providing direct patient consultation outside of breast imaging and IR are still in their infancy stages and only exist as pilot programs or investigational studies. It is therefore hard to assess the effect of the pandemic on these efforts.

#### Beyond the Pandemic—Patient-Centered Radiology

There is emerging literature about radiologists provided consultation and patient access to radiologists. Early studies of direct radiologist-patient communication, have shown favorable and even enthusiastic feedback from patients and improved understanding of the radiologist’s role in their care [8, 47]. Meanwhile, patients are growing accustomed to virtual visits during the pandemic. Subspecialties like breast imaging and IR can benefit from virtual visit workflows and can continue offering these after the pandemic without geographical constraints. For the rest of radiology, the incorporation of virtual patients visits in workflow could become more feasible with less overhead (physical space and staff). Reimbursement for virtual visits, in general, was already becoming an option before the pandemic, but, with the onset of a pandemic, insurers are being required to reimburse for these visits in some states [4]. The implication of that for diagnostic radiology is not yet clear, but, could represent an opportunity to bring radiology closer to patients.

## Closing Remarks on the Virtual Reading Room

While the evolution of PACS and later teleradiology had the undesired side effect of isolating the radiologist from patient care, the virtual radiology reading room offers the potential to make the radiologist more accessible and hence more involved in patient care. It is a modern rethinking of the radiology reading room with the radiology strategic goals and value propositions in mind. The virtual reading room need not imply off-site or on-site coverage; it is rather centered around the idea of creating accessible radiology teams not limited by geographical constraints and operating more efficiently. Existing communication tools are already enabling certain aspects of this vision. The COVID-19 pandemic is forcing many of the elements of the virtual reading room to be developed and adopted. The initial steps were ad hoc in nature due to the rapid onset of the problem. However, now we have the opportunity for a more thoughtful redesign of the reading room that can serve radiology during and beyond the pandemic that would align with its new value propositions. Well-designed informatics solutions are needed to provide a complete integrated system that does not ignore any of the important roles that radiologists play. As is always the case, informatics solutions cannot bring culture changes without a vision and a plan. Procedures and policies will also need to be established to enable the tools to achieve their desired results.
